# Transitions in device and liquid characteristic groupings among US adults frequently using electronic nicotine delivery systems (ENDS) over three timepoints, 2020–2021

**DOI:** 10.18332/tid/171354

**Published:** 2023-10-13

**Authors:** Qinghua Nian, Jeffrey J. Hardesty, Elizabeth Crespi, Joshua K. Sinamo, Ryan D. Kennedy, Kevin Welding, Joanna E. Cohen

**Affiliations:** 1 Institute for Global Tobacco Control, Department of Health, Behavior and Society, Johns Hopkins Bloomberg School of Public Health, Johns Hopkins University, Baltimore, United States

**Keywords:** nicotine concentration, flavor, electronic nicotine delivery system, longitudinal transition patterns, device power

## Abstract

**INTRODUCTION:**

Electronic nicotine delivery system (ENDS) and liquid characteristics affect nicotine and toxicant exposure and use behaviors. Little is known about how adults who frequently use ENDS transition between ENDS device/liquid groupings.

**METHODS:**

A total of 379 US adults (≥21 years) using ENDS frequently (≥5 days/week) self-reported and uploaded photos of their most used ENDS device and liquid in three waves of online surveys (May 2020 – November 2021). Device/liquid grouping was defined by device (i.e. disposable/refillable tank/refillable pod or cartridge/disposable pod or cartridge, adjustable/no adjustable settings) and liquid (i.e. salt/freebase) characteristics. Participants using the same grouping across waves were considered stable users.

**RESULTS:**

The most prevalent wave (W) 1 grouping was tank (freebase, adjustable settings; 36.8%). From W1 to W3, the number of disposable device (salt, no adjustable settings) users increased 156.4% and the number of disposable pod/cartridge (salt, no adjustable settings) users decreased 15.2%. In W2 and W3, compared to stable users, participants using tank (freebase, adjustable settings) in W1 and another grouping in W2 and/or W3 reported significantly higher nicotine concentrations (mg/mL) (W2: 15.1 vs 5.5, p<0.001; W3: 22.9 vs 5.6, p<0.001) and lower device power (watt) (W2: 46.8 vs 58.7, p=0.02; W3: 34.0 vs 57.2, p<0.001).

**CONCLUSIONS:**

Over a 1.5-year period, a rapid growth in disposable device (salt, no adjustable settings) use and a decrease in disposable pod/cartridge (salt, no adjustable settings) use were observed. Participants who transitioned from tank (freebase, adjustable settings) to other groupings were more likely to increase liquid nicotine concentration and reduce device power compared to stable users.

## INTRODUCTION

The US electronic nicotine delivery systems (ENDS) market is characterized by an array of diverse ENDS devices and liquids^1-3^. ENDS can differ in features of the device (disposable, reusable) and liquid storage container (i.e. disposable pod/cartridge, refillable pod/cartridge, tank)^1,2^. Nicotine formulation of the liquid is another varying characteristic, with nicotine salts liquids allowing for smoother delivery of large amounts of nicotine which could improve the sensory experience of vaping^4^. Also, some ENDS devices have added more customizability to the user experience, including adding adjustable settings (e.g. power, airflow, coil) to the devices. These characteristics of ENDS devices and liquids may be associated with different user experiences, use patterns, nicotine content and delivery, flavor preferences, and user characteristics^5^.

A few studies regarding ENDS device use and transition patterns were conducted before 2019. Yingst et al.^6,7^ examined the transition of ENDS use between 2012 and 2014 and found that ENDS users commonly began use with a device shaped like a cigarette and transitioned to a larger device with a more powerful battery that can deliver high levels of nicotine with a wider choice of flavor options and adjustable settings. Felicione et al.^8^ observed the types of ENDS used from 2016 to 2018 in the US, Australia, Canada, and England, and found the most popular ENDS devices in 2018 were refillable tanks (37.3%) and over 80% of ENDS users continued using the same ENDS devices over 18 months.

New ENDS devices on the market and regulatory actions, among other things, may influence people’s ENDS device choices. In January 2020, the United States Food and Drug Administration (FDA) issued a policy prioritizing enforcement against all flavors except menthol and tobacco from disposable podbased ENDS devices such as JUUL, but excluded flavored disposable devices and liquids used in tanks or refillable pods/cartridges^9^. Disposable devices became the most commonly used type of ENDS among adolescents in the US in 2021; these disposable devices typically resemble pod devices in appearance, use high-strength nicotine salt liquid (e.g. Puff Bar with nicotine concentration ranging from 40.6 mg/mL to 52.4 mg/mL) and feature youth-appealing flavors^10,11^. Yingst et al.^12^ conducted a qualitative study to explore the influence of the FDA flavored pod ban on adults (aged ≥21 years) who use JUUL and detected themes about switching from menthol disposable pods to disposable devices that are available in a range of flavors.

Understanding what ENDS devices and liquids people use in recent years, and particularly whether they stably use the same products or whether they change over time, can help inform regulations on ENDS devices and liquids. Most studies related to ENDS use transitions in the US were conducted using data before 2019 and focus on device type without considering other device and liquid characteristics. To fill this gap, we used recent data and incorporated detail on device and liquid characteristics that may affect nicotine content and delivery. The purpose of this study is to explore whether adults who frequently use ENDS changed their most commonly used ENDS device and liquid grouping from 2020 to 2021, and to identify characteristics of devices and liquids that may be associated with such transitions.

## METHODS

### Study sample and protocols

This study used wave 1 (May–October 2020), wave 2 (December 2020–April 2021) and wave 3 (September–November 2021) data of the Vaping and Patterns of E-cigarette Use Research (VAPER) study, a longitudinal cohort study among adults (≥21 years) in the US who used ENDS at least 5 days/week. Participants were recruited using a Craigslist-focused strategy in which ads were posted on the gigs and/or jobs boards in up to 406 locations across the US. To cover well-populated areas, we used US census population estimates for cities and states to identify the frequency of the postings in each Craigslist catchment area. In wave 1, potential participants were considered eligible for this survey if they were aged ≥21 years, used ENDS at least 5 days/week and provided personal identifying information for registration (e.g. name, date of birth, cell phone number, email, and mail address). Eligible participants responded to an online survey hosted by REDCap by reporting their ENDS use patterns and behaviors and submitting photos of their most commonly used ENDS device and most commonly used liquid with that device. Device and liquid characteristics were coded using photos and online searches of manufacturer, academic, retail, and review sites. When device or liquid characteristics were not available from photos or online searches, survey responses were used. Rigorous data review and cleaning procedures were applied upon completion of coding to ensure data quality^13^. The follow-up survey (waves 2 and 3) invitations were sent to those who responded validly and indicated interest in doing the follow-up survey in the previous wave(s). In order to remain eligible in follow-up waves, participants had to report still using ENDS at least 5 days/week.

**Figure 1:**
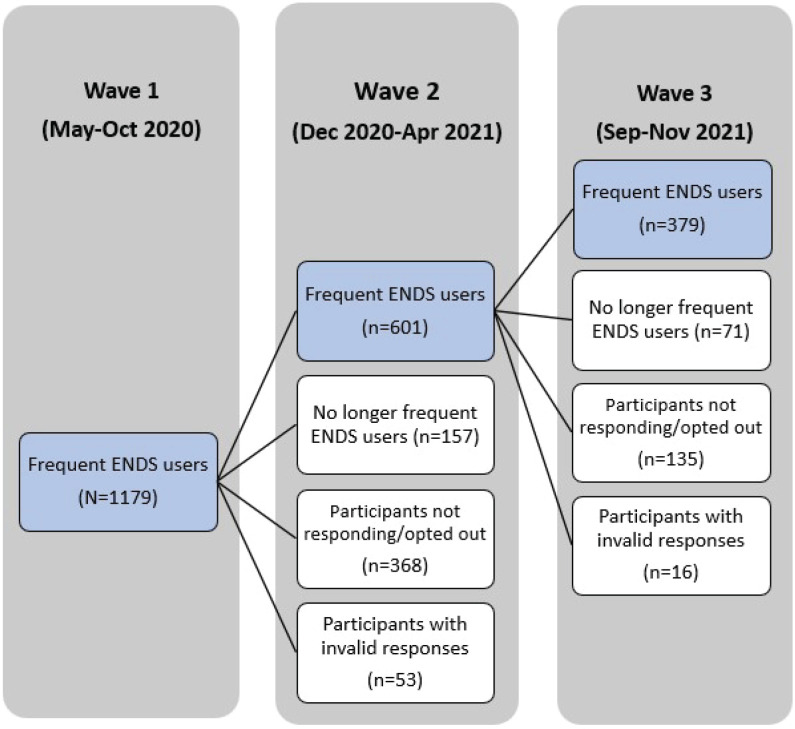
VAPER participants from wave 1 to 3 flowchart, VAPER cohort 1 wave 1–3 study, 2020–2021 (N=1179)

Among 1179 participants who responded validly in wave 1, a total of 601 indicated interest in staying in the study, reported still using ENDS at least five days per week, and provided valid responses in wave 2; among these, 379 reported still using ENDS at least five days per week and provided valid responses in wave 3 (Figure 1). The study sample is the group of participants who responded validly and used ENDS at least five days per week in all three waves (n=379). There was no significant difference between the study sample and other participants in wave 1 in terms of sociodemographic characteristics (including age, gender, race, income, region, and Hispanic origin) and characteristics of device and liquid (including device type, nicotine concentration, and formulation) in wave 1 (p>0.05).

### Measurements

We utilized the approach^14^ proposed to categorize ENDS on the market which examines the combination of ENDS device and liquid characteristics that can affect user nicotine and toxicant exposure. The device/liquid grouping variable describes participants’ most commonly used device (refillable tank; refillable pod or cartridge; disposable pod or cartridge; and disposable device), adjustable/no adjustable settings of device and most commonly used liquid with the device (nicotine salt; freebase). Cartridges and pods are basically the same. Thus, we defined refillable pod or cartridge as refillable cartridge; and disposable pod or cartridge as disposable pod. A device was considered to have adjustable settings if it allows users to modify the power, coil, or airflow. Participants reported using 12 device/liquid groupings in wave 1 with 89.0% using one of five device/liquid groupings, including: 1) tank device with a freebase liquid and adjustable settings (n=139; 36.8%) – GeekVape Aegis Legend device with a freebase liquid was the most commonly reported brand/model; 2) disposable pod device with a nicotine salt liquid and no adjustable settings (n=90; 23.8%) – JUUL was the most commonly reported brand/model; 3) refillable cartridge device with a nicotine salt liquid and adjustable settings (n=51; 13.4%) – SMOK Novo 2 with a nicotine salt liquid was the most commonly reported brand/model; 4) refillable cartridge device with a freebase liquid and adjustable settings (n=41; 10.9%) – SMOK Novo 2 with a freebase liquid was the most commonly reported brand/model; and 5) disposable device with a nicotine salt liquid and no adjustable settings (n=15; 4.1%) – Puff Bar Plus was the most commonly reported brand/model (Supplementary file).

Among participants using these five device/liquid groupings (n=336), participants using the same device/liquid grouping across waves were considered stable users, and participants using one grouping in wave 1 and transitioning to another grouping in waves 2 and/or 3 were considered unstable users.

Device power (wattage) was assessed by examining the device display in the submitted photos if available. Otherwise, several strategies were taken to estimate values of power including calculating the wattage using Ohm’s law via available voltage and resistance data, calculating an adjusted midpoint of the device minimum and maximum wattage based on the average percent of the range utilized by participants for each type and each wave, and using self-reported wattage for wave 3 data, or purchasing and measuring device power using a multimeter.

Nicotine concentration (mg/mL) was based on coding participants’ submitted photos if available. Otherwise, self-report data were used. Nicotine concentration was treated as missing if only self-report data were available and the reported value was >100 mg/mL.

Primary flavor of liquid was assessed by identifying the flavor descriptions on the liquid container of the submitted photos or online searches of the liquid flavor in the photo. Self-report data were used where photo data were missing. We categorized flavors following the ENDS liquid flavor wheel developed by Krüsemann et al.^15^ and then classified the primary flavor into four categories^16^: 1) sweet (including dessert, fruit, candy, other sweets, or other flavors with subcategory listed as sweet); 2) menthol/mint; 3) tobacco; and 4) other (including coffee/tea, nuts, spices, unflavored, multiple flavors, or concept flavor).

Dependence of ENDS was assessed by 4-item E-cigarette Dependence Scale (EDS) scores^17,18^. The score was derived by calculating the mean of the following four items: 1) ‘I find myself reaching for my e-cigarette without thinking about it’, 2) ‘I drop everything to go out and buy e-cigarettes or e-juice’, 3) ‘I vape more before going into a situation where vaping is not allowed, and 4) ‘When I haven’t been able to vape for a few hours, the craving gets intolerable’. Response options were: 0 ‘never’, 1 ‘rarely’, 2 ‘sometimes’, 3 ‘often’, and 4 ‘almost always’. A greater EDS score is indicative of a greater level of ENDS use dependence.

Sociodemographic variables based on self-report data include geographical location (Northeast, Midwest, South, West), gender (male, female, non-male/female, prefer not to answer), age (21–24, 25–29, 30–44, 45–54, 55–69), income ($) (0–39999, 40000–59999, 60000–99999, ≥100000, prefer not to answer), race (White, non-White or multi-racial, prefer not to answer), ethnicity (Hispanic/Latino, non-Hispanic/Latino), sexual identity (heterosexual or straight, non-heterosexual or straight, prefer not to answer), and disability (yes, no, prefer not to answer) which was measured by whether people were limited in the kind or amount of work they can do because of a physical, mental or emotional problems. Smoking status in wave 1 was assessed by whether people had smoked a cigarette in the past 30 days.

### Statistical analysis

The distribution of participants’ sociodemographic characteristics was generally similar to that of daily ENDS users in the 2019 Tobacco Use Supplement to the Current Population Survey (TUS-CPS)^13^; nevertheless, post-stratification survey weighting for gender/age/race was applied based on data from the 2019 TUS-CPS to ensure representativeness to US adult frequent ENDS users. Descriptive statistics across sociodemographic variables, characteristics of device and liquid, and ENDS dependence were provided for the overall sample and sample by device/liquid grouping and transition. Rao-Scott chi-squared tests were used to evaluate differences in categorical variables. Independent t-tests and one-way ANOVA were used to test for differences in continuous variables. Non-parametric methods, including Mann-Whitney U and the Kruskal-Wallis H, were used when the sample size was <30. We used survey weights for all analyses and reported weighted frequencies scaled to our sample size, and p-values adjusted to account for the large sample size resulting from the implementation of the survey weights. The pairwise deletion method was used to deal with the missing data (5.5% of the total participants). All analyses were conducted using SAS (Version 9.4, SAS Institute, Cary, NC). A 2-sided p<0.05 was used to determine statistical significance.

## RESULTS

The majority (76.5%) of the participants were aged <45 years, White (89.7%), and non-Hispanic (92.4%); 41.6% had an annual income <$40000 (Table 1). Approximately 30% of participants (29.8%) reported that they had smoked a cigarette in the past 30 days in wave 1.

**Table 1:** Sociodemographic characteristics of participants, by the most common device/liquid groupings^a^, VAPER cohort 1 wave 1–3 study, 2020–2021 (N=379)

*Characteristics*	*Categories*	*Total (N=379)*	*Refillable tank (freebase, adjustable settings) (N=139)*	*Refillable cartridge (salt, adjustable settings) (N=51)*	*Refillable cartridge (freebase, adjustable settings) (N=41)*	*Disposable pod (salt, no adjustable settings) (N=90)*	*Disposable device (salt, no adjustable settings) (N=15)*
		*n (%)*	*n (%)*	*n (%)*	*n (%)*	*n (%)*	*n (%)*
**Geographical location** (p=0.62)	Northeast	33 (8.8)	13 (9.4)	5 (9.1)	1 (2.4)	12 (13.0)	1 (6.4)
	Midwest	70 (18.5)	21 (15.3)	7 (13.7)	9 (22.7)	23 (25.0)	2 (11.3)
	South	165 (43.4)	63 (45.0)	24 (47.1)	19 (46.6)	34 (37.7)	7 (42.3)
	West	111 (29.3)	42 (30.3)	15 (30.2)	12 (28.4)	22 (24.2)	6 (40.0)
**Age** (years) (p<0.001*)	21–29	84 (22.1)	21 (14.8)	20 (38.3)	4 (9.0)	25 (27.2)	7 (43.1)
	30–44	206 (54.4)	92 (66.3)	25 (48.7)	24 (57.2)	43 (48.1)	5 (34.6)
	≥45	89 (23.5)	26 (18.9)	7 (13.0)	14 (33.8)	22 (24.8)	3 (22.3)
**Gender** (p=0.24)	Male	191 (50.3)	80 (57.1)	28 (54.1)	18 (42.8)	40 (44.9)	6 (37.2)
	Female	185 (48.9)	59 (42.2)	22 (43.9)	24 (57.2)	49 (54.0)	10 (62.8)
	Other	2 (0.5)	1 (0.7)	0 (0.0)	0 (0.0)	1 (1.1)	0 (0.0)
	Prefer not to answer	1 (0.3)	0 (0.0)	1 (1.9)	0 (0.0)	0 (0.0)	0 (0.0)
**Race** (p=0.09)	White	340 (89.7)	125 (90.0)	43 (84.3)	38 (92.7)	81 (90.0)	12 (80.0)
	Other or multi race	35 (9.3)	12 (8.5)	7 (13.7)	3 (7.3)	8 (8.9)	3 (20.0)
	Prefer not to answer	4 (1.0)	2 (1.5)	1 (2.0)	0 (0.0)	1 (1.1)	0 (0.0)
**Hispanic origin** (p=0.75)	No	350 (92.4)	128 (91.5)	48 (94.1)	37 (89.8)	85 (94.1)	13 (86.5)
	Yes	25 (6.6)	10 (7.4)	2 (4.5)	3 (7.9)	5 (5.4)	2 (13.5)
	Prefer not to answer	4 (1.0)	2 (1.1)	1 (1.3)	1 (2.4)	1 (0.6)	0 (0.0)
**Annual household income** ($) (p=0.52)	<40000	158 (41.6)	58 (41.6)	23 (45.4)	19 (45.0)	35 (39.0)	6 (38.9)
	40000–59999	99 (26.0)	38 (27.1)	9 (16.9)	13 (31.8)	21 (23.6)	3 (18.8)
	60000– 99999	74 (19.5)	29 (20.8)	9 (18.6)	4 (8.7)	22 (24.8)	5 (30.8)
	≥100000	40 (10.6)	9 (6.4)	9 (17.5)	5 (12.2)	11 (12.0)	1 (8.1)
	Prefer not to answer	9 (2.2)	6 (4.1)	1 (1.6)	1 (2.4)	1 (0.6)	1 (3.3)
**Sexual identity** (p=0.08)	Heterosexual or straight	294 (77.4)	110 (78.7)	41 (80.1)	26 (62.1)	68 (75.0)	15 (94.7)
	Other	78 (20.5)	25 (17.9)	9 (18.3)	16 (37.9)	20 (22.2)	1 (5.3)
	Prefer not to answer	8 (2.1)	5 (3.4)	1 (1.6)	0 (0.0)	2 (2.8)	0 (0.0)
**Disability** (p=0.22)	No	280 (73.8)	101 (72.4)	43 (84.8)	29 (71.0)	65 (72.6)	14 (90.4)
	Yes	87 (23.0)	34 (24.4)	6 (12.0)	10 (24.7)	24 (26.8)	1 (9.6)
	Prefer not to answer	12 (3.1)	4 (3.2)	2 (3.2)	2 (4.3)	1 (0.6)	0 (0.0)
**Smoking status in wave 1** (p=0.02*)	No	266 (70.2)	111 (79.7)	33 (64.2)	32 (77.7)	56 (62.0)	9 (56.8)
	Yes	113 (29.8)	28 (20.3)	18 (35.8)	9 (22.3)	34 (38.0)	7 (43.2)

a The most common device/liquid groupings reported were about the groupings that participants used in wave 1. The Rao-Scott chi-squared test was not computed for age, gender, race and Hispanic origin by device/liquid grouping because at least one table cell had 0 frequency (e.g. reported gender as other or prefer not to answer); we tested the differences by excluding ‘prefer not to answer’ responses.

* Significant at p<0.05 level.

Users of these 5 groupings had similar characteristics with respect to geographical region, gender, race, Hispanic origin, annual household income, sexual identity, and disability (Table 1). Significantly greater percentages of participants who used a disposable device (nicotine salt, no adjustable settings) were aged <30 years (43.1%) and smoked in the past 30 days in wave 1 (43.2%) compared to participants who used a refillable cartridge device (freebase, adjustable settings) (9.0% and 22.3%, respectively, p<0.05) and participants who used a tank device (freebase, adjustable settings) (14.8% and 20.3%, respectively, p<0.05).

**Figure 2:**
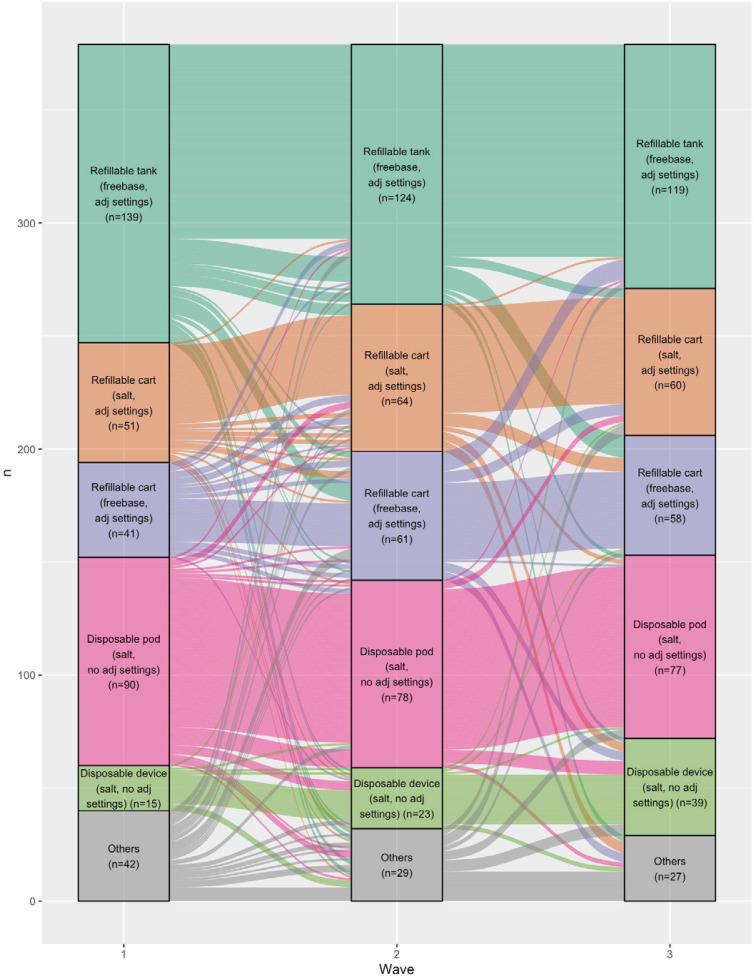
Transition in device/liquid grouping among participants from wave 1 to wave 3, VAPER cohort 1 wave 1–3 study, 2020–2021 (N=379)

From wave 1 to wave 3, the total number of tank device (freebase, adjustable settings) users and disposable pod device (nicotine salt, no adjustable settings) users decreased 14.7% and 15.2%, respectively; and the total number of refillable cartridge device (nicotine salt, adjustable settings), refillable cartridge device (freebase liquid, adjustable settings) and disposable device (nicotine salt, no adjustable settings) users increased 16.8%, 40.1% and 156.4%, respectively (Figure 2).

Over 60% of participants who used a disposable pod device (nicotine salt, no adjustable settings) (71.5%), re-useable tank device (freebase, adjustable settings) (68.2%), refillable cartridge device (nicotine salt, adjustable settings) (65.0%), and disposable device (nicotine salt, no adjustable settings) (64.0%) in wave 1 and less than half of participants who used refillable cartridge device (freebase liquid, adjustable settings) (45.9%) in wave 1, reported using the same device/liquid groupings across three waves (Table 2).

**Table 2:** Differences between participants who stably used the same device/liquid grouping and participants transitioned from wave 1 to wave 3 by device/liquid grouping, VAPER cohort 1 wave 1–3 study, 2020–2021 (N=379)

	*Total (N=379)*	*Refillable tank (freebase, adjustable settings) (N=139)*		*Refillable cartridge (salt, adjustable settings) (N=51)*		*Refillable cartridge (freebase, adjustable settings) (N=41)*		*Disposable pod (salt, no adjustable settings) (N=90)*		*Disposable device (salt, no adjustable settings) (N=15)*	
		*Stable users*	*Unstable users*	*Stable users*	*Unstable users*	*Stable users*	*Unstable users*	*Stable users*	*Unstable users*	*Stable users*	*Unstable users*
		*(n=95)*	*(n=45)*	*(n=33)*	*(n=18)*	*(n=19)*	*(n=23)*	*(n=64)*	*(n=26)*	*(n=10)*	*(n=6)*
**Nicotine concentration**	M (SE)	M (SE)	M (SE)	M (SE)	M (SE)	M (SE)	M (SE)	M (SE)	M (SE)	M (SE)	M (SE)
**Wave 1 (p<0.001)**	23.5 (1.1)	5.5 (0.5)	6.5 (0.6)	37.0 (2.0)	37.5 (4.5)	6.6 (1.2)	9.0 (1.3)	46.9 (2.1)	45.5 (3.0)	50.9 (0.6)	50.0 (0.0)
		p=0.19		p=0.89		p=0.16		p=0.68		p=0.31	
**Wave 2 (p<0.001)**	24.4 (1.2)	5.5 (0.5)	15.1 (2.7)	36.9 (2.4)	29.7 (5.7)	6.6 (1.0)	15.0 (3.3)	48.5 (1.4)	35.2 (4.6)	50.0 (1.2)	51.5 (0.9)
		p<0.001*		p=0.23		p=0.02*		p<0.01*		p=0.39	
**Wave 3 (p<0.001)**	25.0 (1.2)	5.6 (0.5)	22.9 (3.4)	33.3 (2.8)	24.6 (5.1)	7.4 (1.3)	19.3 (3.9)	47.5 (1.9)	33.7 (4.8)	50.0 (0.0)	50.7 (0.7)
		p<0.001*		p=0.09		p<0.01*		p<0.01*		p=0.36	
**Primary flavors**	n (%)	n (%)	n (%)	n (%)	n (%)	n (%)	n (%)	n (%)	n (%)	n (%)	n (%)
**Wave 1**											
Sweet	227 (65.8)	80 (85.4)	36 (80.6)	24 (73.4)	14 (79.9)	14 (76.4)	17 (73.7)	1 (2.5)	2 (12.3)	9 (96.4)	6 (100.0)
Menthol/Mint	46 (13.2)	1 (0.9)	3 (6.1)	4 (12.8)	1 (1.9)	1 (5.2)	2 (8.6)	27 (54.2)	7 (46.0)	1 (3.6)	0 (0.0)
Tobacco	44 (12.6)	4 (3.8)	1 (2.2)	3 (8.4)	1 (5.4)	3 (16.6)	1 (4.3)	21 (43.3)	6 (38.5)	0 (0.0)	0 (0.0)
Other	29 (8.4)	9 (9.9)	5 (11.1)	2 (5.5)	2 (12.8)	1 (1.8)	3 (13.4)	0 (0.0)	1 (3.1)	0 (0.0)	0 (0.0)
		p=0.53		p=0.63		p=0.85		p=0.13		N/A	
**Wave 2**											
Sweet	223 (63.9)	75 (85.5)	39 (85.6)	22 (71.7)	11 (66.1)	15 (80.7)	15 (67.2)	1 (1.6)	11 (43.7)	10 (100.0)	4 (66.5)
Menthol/Mint	54 (15.5)	0 (0.0)	2 (5.4)	3 (8.5)	0 (0.0)	1 (5.2)	1 (4.5)	29 (55.8)	8 (34.4)	0 (0.0)	2 (33.5)
Tobacco	40 (11.5)	4 (5.0)	1 (2.2)	3 (8.5)	0 (0.0)	1 (1.8)	3 (14.3)	22 (42.6)	4 (17.9)	0 (0.0)	0 (0.0)
Other	32 (9.2)	8 (9.5)	3 (6.7)	3 (11.3)	6 (33.9)	2 (12.2)	3 (14.0)	0 (0.0)	1 (4.0)	0 (0.0)	0 (0.0)
		p=0.98		p=0.70		p=0.32		p<0.001*		N/A	
**Wave 3**											
Sweet	230 (65.3)	76 (86.7)	37 (81.9)	24 (72.2)	15 (82.1)	14 (77.8)	16 (75.5)	0 (0.0)	17 (64.4)	8 (87.7)	1 (27.0)
Menthol/Mint	56 (16.0)	1 (1.1)	3 (6.1)	6 (19.8)	1 (6.4)	3 (15.0)	2 (9.0)	30 (57.7)	3 (11.6)	0 (0.0)	1 (16.8)
Tobacco	40 (11.5)	4 (4.1)	0 (1.1)	3 (8.0)	0 (0.0)	1 (7.2)	3 (11.8)	22 (42.3)	3 (10.7)	0 (0.0)	0 (0.0)
Other	26 (7.3)	7 (8.1)	5 (10.9)	0 (0.0)	2 (11.5)	0 (0.0)	1 (3.8)	0 (0.0)	3 (13.3)	1 (12.3)	3 (56.2)
		p=0.53		p=0.44		p=0.87		N/A		p<0.001*	
**Wattage**	M (SE)	M (SE)	M (SE)	M (SE)	M (SE)	M (SE)	M (SE)	M (SE)	M (SE)	M (SE)	M (SE)
**Wave 1 (p<0.001)**	34.0 (1.6)	61.5 (2.9)	56.4 (3.8)	17.1 (1.0)	16.3 (1.1)	19.1 (1.9)	21.7 (4.4)	13.0 (0.4)	12.5 (0.8)	9.6 (0.1)	9.4 (0.0)
		p=0.27		p=0.61		p=0.51		p=0.52		p=0.31	
**Wave 2 (p<0.001)**	33.0 (1.6)	58.7 (2.9)	46.8 (5.2)	16.3 (1.5)	21.8 (4.5)	18.8 (2.2)	35.5 (7.3)	13.5 (0.4)	14.9 (1.4)	10.8 (1.0)	14.6 (2.8)
		p=0.02*		p=0.24		p=0.03*		p=0.30		p=0.30	
**Wave 3 (p<0.001)**	31.4 (1.5)	57.2 (2.7)	34.0 (4.4)	16.4 (1.3)	28.0 (8.1)	20.5 (2.1)	26.0 (5.5)	13.6 (0.4)	20.5 (3.5)	12.1 (0.8)	13.0 (0.8)
		p<0.001*		p=0.13		p=0.30		p=0.04*		p=0.46	
**ENDS dependence (0–4, 4 being the most dependent)**	M (SE)	M (SE)	M (SE)	M (SE)	M (SE)	M (SE)	M (SE)	M (SE)	M (SE)	M (SE)	M (SE)
**Wave 1 (p<0.001)**	2.3 (0.1)	2.2 (0.1)	2.1 (0.2)	2.2 (0.2)	2.7 (0.2)	1.8 (0.2)	2.6 (0.2)	2.3 (0.1)	2.3 (0.2)	2.4 (0.3)	3.2 (0.3)
		p=0.78		p=0.06		p<0.01*		p=0.98		p=0.051	
**Wave 2 (p<0.001)**	2.4 (0.1)	2.2 (0.1)	2.2 (0.1)	2.4 (0.2)	2.6 (0.2)	2.0 (0.2)	2.6 (0.1)	2.5 (0.1)	2.6 (0.2)	2.5 (0.3)	2.8 (0.6)
		p=1.00		p=0.35		p<0.01*		p=0.72		p=0.52	
**Wave 3 (p<0.001)**	2.4 (0.0)	2.2 (0.1)	2.3 (0.2)	2.4 (0.1)	2.8 (0.2)	2.1 (0.2)	2.6 (0.1)	2.5 (0.1)	2.6 (0.2)	3.1 (0.2)	2.9 (0.5)
		p=0.68		p=0.07		p=0.02*		p=0.54		p=0.76	

The Rao-Scott chi-squared test was not computed for primary flavor because at least one table cell had 0 frequency; we tested the differences using the records with flavor as sweet or other flavors.

* Significant at p<0.05 level. M: mean. SE: standard error.

There was no significant difference between stable and unstable users of each device/liquid grouping in nicotine concentration, primary flavor of liquid and device power in wave 1 (Table 2). Among participants who used a tank device (freebase, adjustable settings) in wave 1, compared to unstable users, the stable users used liquid with significantly lower nicotine concentration (W2: 5.5 vs 15.1, p<0.001; W3: 5.6 vs 22.9, p<0.001) and device with significantly higher power (W2: 58.7 vs 46.8, p=0.02; W3: 57.2 vs 34.0, p<0.001) in waves 2 and 3. Among participants who used a disposable pod device (nicotine salt, no adjustable settings) in wave 1, compared to unstable users, stable users reported using liquid with significantly greater nicotine concentration (W2: 48.5 vs 35.2, p<0.01; W3: 47.5 vs 33.7, p<0.01) in waves 2 and 3 and device with significantly lower power (W3: 13.6 vs 20.5, p=0.04) in wave 3. In addition, significantly greater percentages of stable users of this grouping reported using menthol/mint (57.7%) or tobacco (42.3%) flavored liquids compared to unstable users (11.6% and 10.7%, respectively; p<0.01) in wave 3. Among participants who used a refillable cartridge device (freebase liquid, adjustable settings) in wave 1, compared to unstable users, stable users reported using liquid with significantly lower nicotine concentration (W2: 6.6 vs 15.0, p=0.02; W3: 7.4 vs 19.3, p<0.01) in waves 2 and 3 and device with significantly higher power (W2: 18.8 vs 35.5, p=0.03) in wave 2.

With respect to ENDS dependence, no significant differences were found between stable and unstable users except those who used a refillable cartridge (freebase, adjustable settings) in wave 1. Stable users of that grouping reported significantly lower ENDS dependence in three waves (W1: 1.8 vs 2.6, p<0.01; W2: 2.0 vs 2.6, p=0.01; W3: 2.1 vs 2.6, p=0.02) compared to unstable users (Table 2).

## DISCUSSION

Using a longitudinal survey design, this study explored how frequent ENDS users transitioned between various ENDS device/liquid groupings and examined the association between such transitions and characteristics of device and liquids and ENDS dependence. In wave 1, approximately 90% of frequent ENDS users used one of five device/liquid groupings and more than one-third of frequent ENDS users reported using tank device (freebase, adjustable settings). The majority of frequent ENDS users reported using the device/liquid grouping stably across waves, except users of refillable cartridge device (freebase, adjustable settings). These findings provide more detailed information about transition by examining a combination of device and liquid characteristics compared to the previous literature that has examined device type and liquid separately^6-8^.

Since 2020, the sales of disposable pods/cartridges (such as JUUL) have declined, while the market for disposable devices (such as Elf Bar) continues to grow through the beginning of 2023^19^. In 2021, disposable devices surpassed disposable pods as the most used type of ENDS among adolescents^11,20^. We also observed a 15.2% decrease in users of disposable pods/cartridges (10.8% disposables, 4.4% other) and a 156.4% increase in users of disposable devices from May 2020 to November 2021. The transition from disposable pods/cartridges to other devices may be related to the loophole in the FDA flavor decision in 2020 which only bans flavored disposable pod ENDS products (other than tobacco- or menthol-flavored) and excludes the flavored disposable devices and liquids used in tanks or refillable pods/cartridges^10,12,21,22^. Sweet flavors were the most popular among all ENDS users in this study except those who used a disposable pod device (nicotine salt, no adjustable settings) that is covered by the FDA 2020 flavor decision. Further, about two-thirds of users (64.4%) who transitioned from disposable pod to other groupings used sweet flavors in wave 3. Ali et al.^23^ conducted a study in Massachusetts, New York, Rhode Island, and Washington, and found that banning sale of flavored ENDS in these states was associated with a reduction in total ENDS sales.

Also, the choice of ENDS device/liquid grouping in wave 1 was significantly correlated with frequent ENDS users’ age and smoking status in the past 30 days. ENDS users who were aged <30 years or smoked in the past 30 days were more likely to use disposable devices (nicotine salt, no adjustable settings) and less likely to use tank devices (freebase, adjustable settings) or refillable cartridge device (freebase, adjustable settings). Previous studies found that ENDS users who used disposable devices were less likely to quit smoking compared to those who used tank devices^24^. Continued efforts are needed to track the use of disposable and other types of devices among dual users so we can understand how various ENDS device/liquid groupings positively and negatively affect cigarette use patterns.

ENDS users who transitioned from tank device (freebase, adjustable settings) and refillable cartridge (freebase, adjustable settings) to other groupings were more likely to increase nicotine concentration of their liquid, while ENDS users who transitioned from disposable device (nicotine salt, no adjustable settings) to other groupings continued to use high nicotine concentration around 50 mg/mL. Among tank device (freebase, adjustable settings) users in wave 1 who transitioned to other device/liquid groupings, nicotine concentration of their liquid significantly increased from 6.5 to 22.9 mg/mL on average and power of their device significantly decreased from 56.4 to 34.0 watt, on average. These findings may show a trend among ENDS users towards high nicotine concentrations. Previous studies found that US ENDS users were more likely to increase nicotine concentration from <20 to ≥21 mg/mL compared to ENDS users in other countries^8^. Unlike some countries, such as England, who imposed an upper limit of 20 mg/mL, the US does not restrict the nicotine concentration of ENDS liquid^8^. The significant increase in using disposable devices and transition to devices that contain nicotine salts and liquid with higher nicotine concentration may lead to an increased dependence on ENDS. Furthermore, ENDS device power also has an influence on nicotine emission and delivery^25,26^. Although tank devices are often paired with relatively lower nicotine concentration liquids, tank devices (freebase, adjustable settings) have the highest power level among all device/liquid groupings. Higher powered ENDS devices have a larger range of nicotine delivery potential^27,28^. To regulate nicotine delivery of ENDS, various device specifications and liquid characteristics, such as device power and nicotine formulation and concentration, need to be considered.

The longitudinal data presented are from a recent sample across the US that reflect ENDS devices and liquids people use under the rapidly evolving ENDS market and recent regulations. Additionally, post-stratification weights ranging from 0.64 to 1.56 were applied to improve representativeness. The data quality and quantity is optimized by employing a range of data integrity procedures and by using photo data of devices and liquids^13,29^. We examined the transition of ENDS device/liquid grouping rather than device type alone, which allows exploring key variability influencing nicotine delivery^14,30^.

### Limitations

The data were collected in the US during the COVID pandemic, which may have influenced people’s ENDS use behaviors. The findings likely have limited generalizability to other countries due to the current market and regulatory context in the US. The sample size of disposable device users in wave 1 was small (n=15); thus, non-parametric methods were used to test the hypotheses for this group. Further, all results were based on unadjusted analyses. Future studies could examine transition patterns and correlates of transitions in more depth with a larger sample and an adjusted analysis to control for confounding. Another limitation is that data reported are about participants’ most commonly used device and most commonly used liquid with that device; participants may use other device(s) and/or liquid(s). Detailed data on additional devices or liquids were not collected to keep the survey to a manageable length for participants and due to resource and time constraints in checking the validity and the coding of the data. This study examined the nicotine concentration, primary flavor, and device power separately. Future research can explore the effect of nicotine delivery and exposure on transition by examining the combination of these variables and puff behavior (e.g. nicotine flux)^31^, which can provide more evidence to inform regulatory efforts on ENDS. In addition, the study focused on people who frequently use ENDS. Our analysis did not account for people who may have used two or more tobacco products (dual or poly tobacco use). Understanding how dual/poly use affects transitions between device/liquid groupings can be the subject of future work.

## CONCLUSIONS

This study used longitudinal data at three time points from 2020 to 2021 to provide evidence of transitions across ENDS device/liquid groupings within a changing marketplace and under the influence of new regulatory actions. Over a 1.5-year period, most frequent ENDS users continued using the same device/liquid groupings except refillable cartridge device (freebase, adjustable settings) users. The number of disposable device (nicotine salt, no adjustable settings) users increased rapidly. Tank device (freebase, adjustable settings) and refillable pod/cart (freebase, adjustable settings) users who transitioned to other devices reported a significant increase in nicotine concentration. In response to device/liquid grouping transition, future research can examine possible changes in health outcomes and nicotine flux to evaluate the public health implications of these behaviors, to identify loopholes that may undermine FDA’s efforts to reduce harms of ENDS use, and to better forecast the potential impacts of possible regulations on nicotine formulation and concentration, and device power.

## Data Availability

The data supporting this research are available from the authors on reasonable request.
