# Supplementary material for: Transitions in device and liquid characteristic groupings among US adults frequently using electronic nicotine delivery systems (ENDS) over three timepoints, 2020–2021
Source: Tob Induc Dis. 2023 Oct 13;21:134. doi: 10.18332/tid/171354 (PMC10571097; doi:10.18332/tid/171354)
Supplement: Supplementary file 1 [file TID-21-134-s1.pdf]

Appendix Table 1. VAPER participants in wave 1 by device/liquid grouping, VAPER cohort 1 wave 1-3 study, 2020-2021 (n=379).

Device/Liquid Grouping in Wave 1	n
Refillable tank (adjustable settings, freebase)	139
Disposable pod (non-adjustable settings, nicotine salt)	90
Refillable cartridge (adjustable settings, nicotine salt)	51
Refillable cartridge (adjustable settings, freebase)	41
Disposable device (non-adjustable settings, nicotine salt)	15
Refillable tank (adjustable settings, nicotine salt)	7
Refillable cartridge (non-adjustable settings, nicotine salt)	6
Disposable pod (non-adjustable settings, freebase)	4
Disposable device (adjustable settings, nicotine salt)	1
Refillable cartridge (non-adjustable settings, freebase)	1
Disposable pod (adjustable settings, nicotine salt)	1
Disposable device (non-adjustable settings, freebase)	1
Missing	21

Appendix Table 2. Brands/models reported in wave 1 by device/liquid grouping, VAPER cohort 1 wave 1-3 study, 2020-2021 (n=*3*79).

Device/Liquid Grouping	N of Brands/Models	Most Commonly Reported Brands/Models
Refillable tank (adjustable settings, freebase)	70	GeekVape Aegis Legend (n=18, 13%)
Disposable pod (non-adjustable settings, nicotine salt)	6	JUUL (n=43, 47%)
Refillable cartridge (adjustable settings, nicotine salt)	20	SMOK Novo 2 (n=14, 39%)
Refillable cartridge (adjustable settings, freebase)	23	SMOK Novo 2 (n=4, 10%)
Disposable device (non-adjustable settings, nicotine salt)	9	Puff Bar Plus (n=6, 39%)

© 2023 Nian Q. et al.
